# Supplementary material for: Multi-omics profiling of younger Asian breast cancers reveals distinctive molecular signatures
Source: Nat Commun. 2018 Apr 30;9:1725. doi: 10.1038/s41467-018-04129-4 (PMC5928087; doi:10.1038/s41467-018-04129-4)
Supplement: Supplementary file 1 — Supplementary Information [file 41467_2018_4129_MOESM1_ESM.pdf]

Supplementary Information

Multi-omics Profiling of Younger Asian Breast Cancers Reveals Distinctive Molecular Signatures

Kan et al.

Supplementary Figure 1

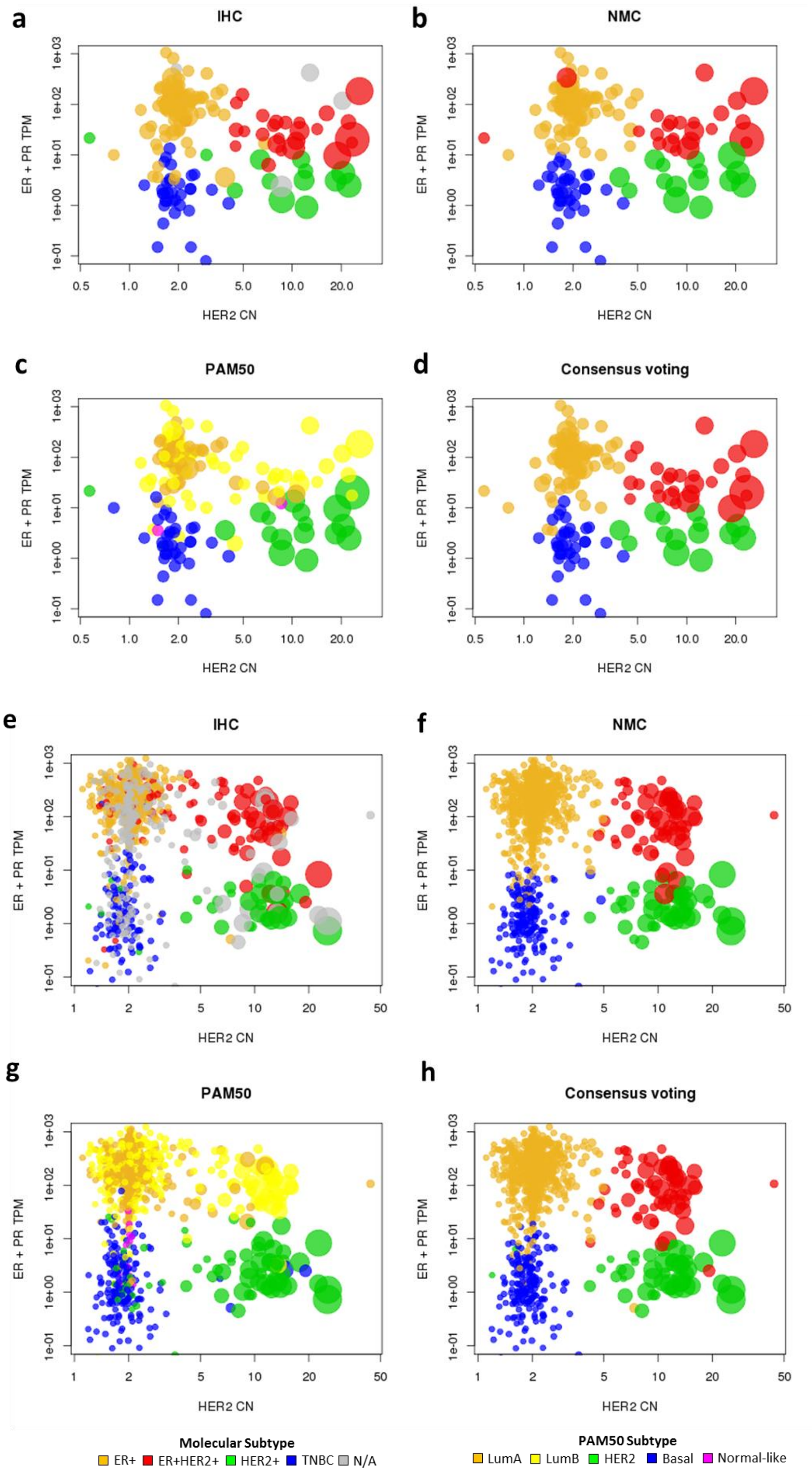

Supplementary Figure 1: Molecular subtype classification. Molecular subtype classifications based on the IHC (a, e), NMC (b, f), PAM50 (c, g) and Consensus (d, h) methods for SMC (a-d) and TCGA (e-h). X-axis of the scatter plot represents copy number of HER2 (*ERBB2*) in log2 scale while Y-axis represents the average expression of ER (*ESR1*) and PR (*PGR*) in TPM. Dot sizes are proportional to HER2 gene expression.

Supplementary Figure 2

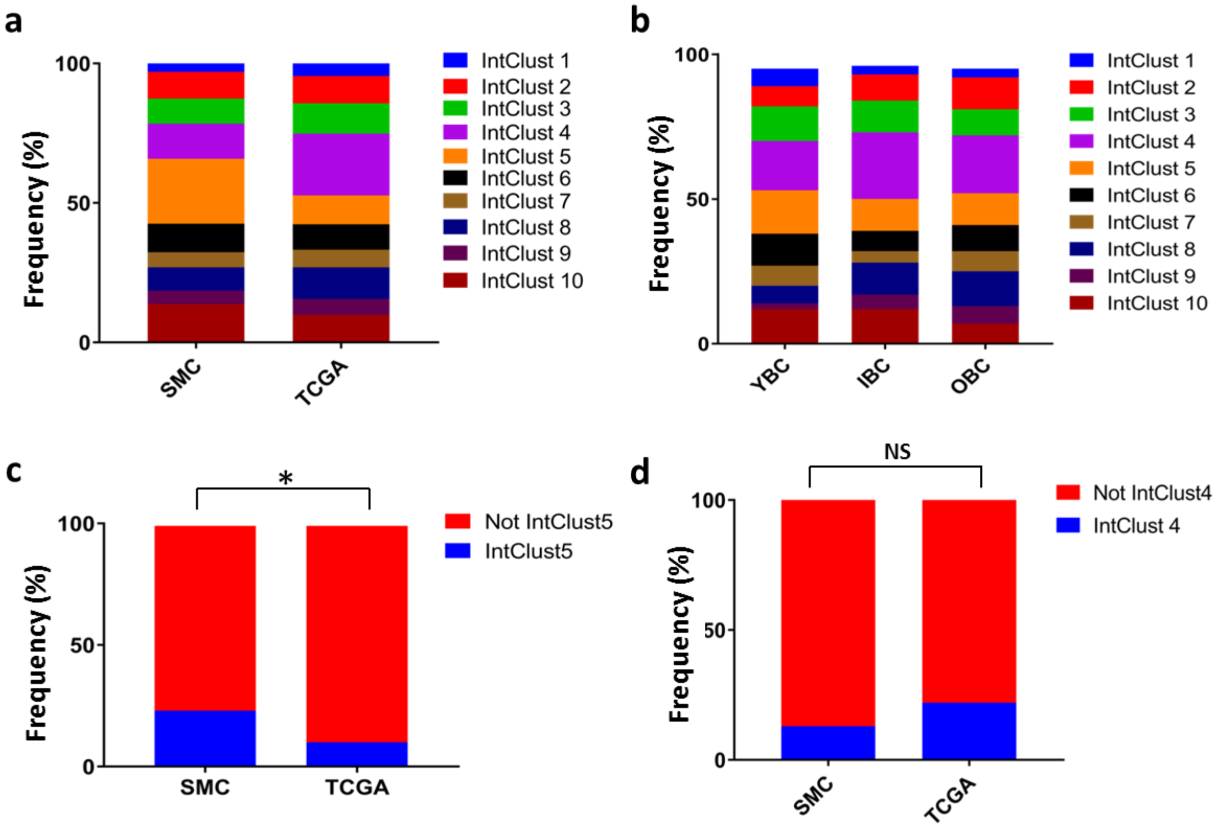

Supplementary Figure 2: Integrated genomic subtype classification. Stacked bar charts depicting the sample distributions in (a) IntClust subtypes across different cohorts, (b) IntClust subtypes across three age groups, (c) IntClust 5 and (d) IntClust 4 across different cohorts. Chi-squared analysis was used to test IntClust enrichment across cohorts and age groups in a-b; Fisher's exact test was used to determine enrichment of IntClust 5 and 6 across cohorts in figure c-d: * $p < 0.05$; NS: $p \geq 0.05$.

Supplementary Figure 3

a

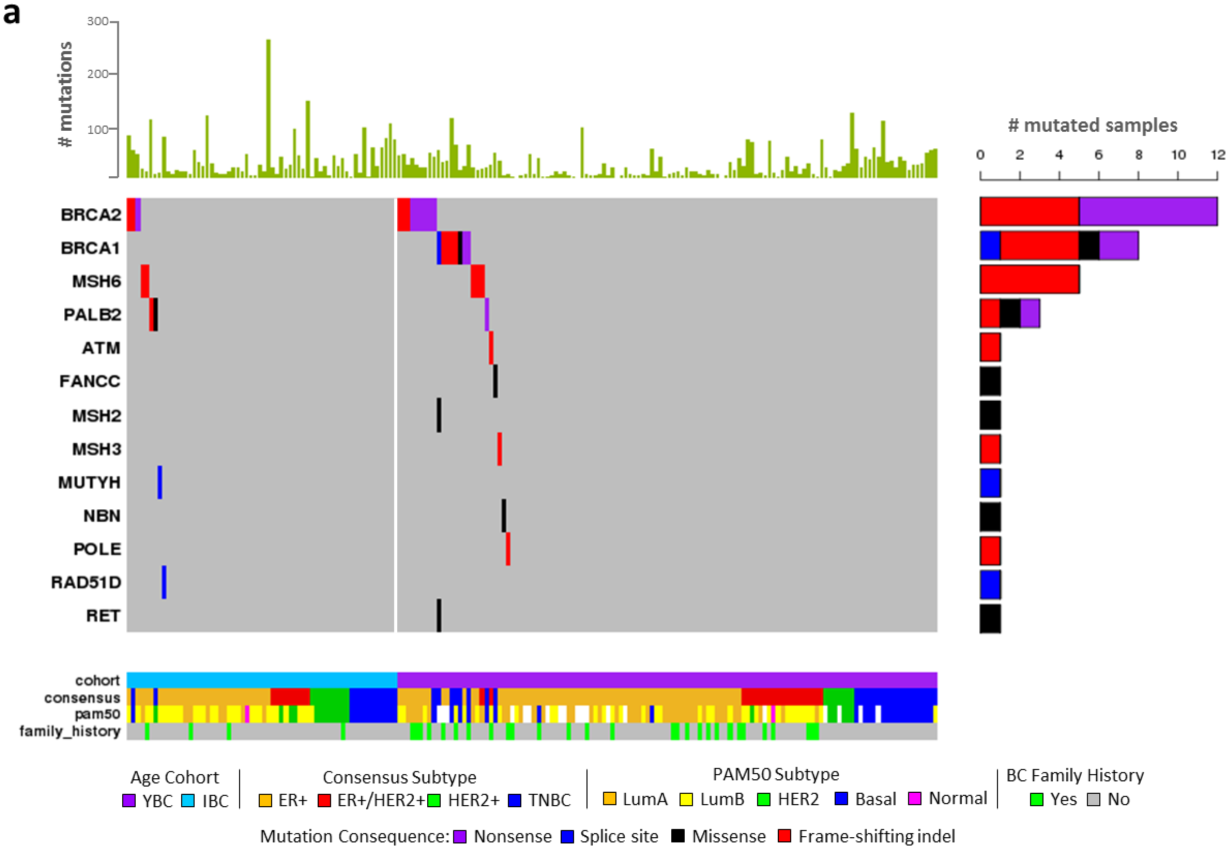

b

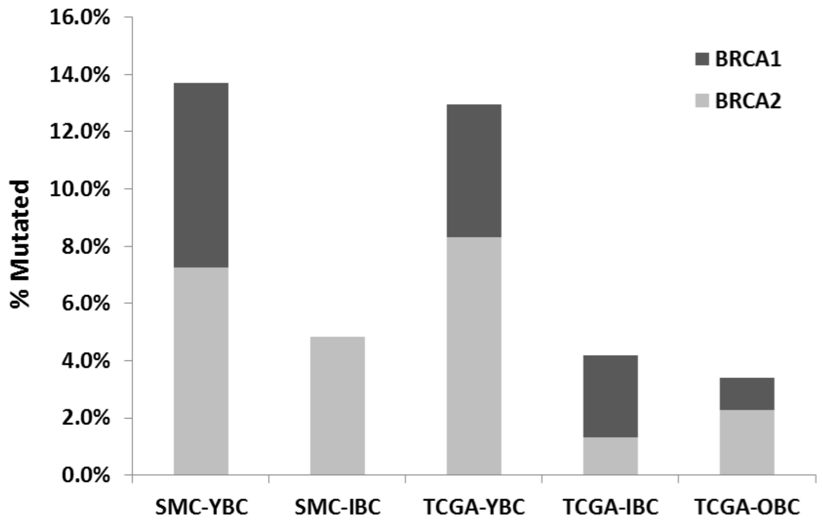

Supplementary Figure 3: Landscape of germline variants in breast cancer predisposition genes. (a) Shown in the heatmap are pathogenic germline mutations identified in 13 breast cancer susceptibility genes (rows) and all SMC samples (columns), color coded by mutation consequences. White vertical line demarcates YBC on the left and OBC samples on the right. The bar chart above represents the sample-level count of protein-altering somatic mutations. The stacked bar chart to the right illustrates the gene-level prevalence of germline mutations. Column color labels represent age-based group (Cohort), intrinsic molecular subtype classifications (Consensus and PAM50) and breast cancer family history (Family history) statuses for all samples. (b) *BRCA1*/*BRCA2* germline pathogenic mutation frequencies in different age groups of SMC and TCGA.

Supplementary Figure 4

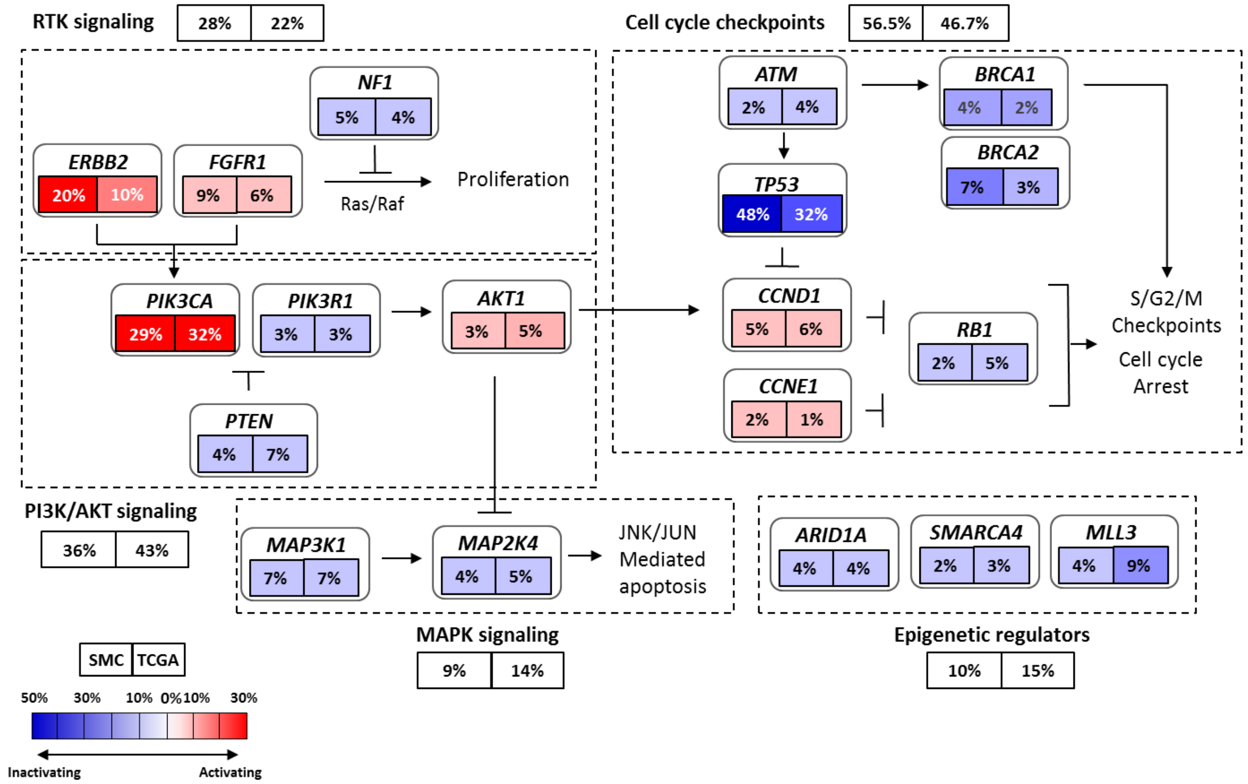

Supplementary Figure 4: Frequently altered oncogenic pathways. In this diagram, genes in the same pathways are connected by arrows and enclosed in the dotted outline. Alteration prevalence for each gene in SMC (left) and TCGA (right) is denoted by the numerical values and represented by the color gradient. Genes are further differentiated by color based on whether the majority of alterations are inactivating or activating.

Supplementary Figure 5

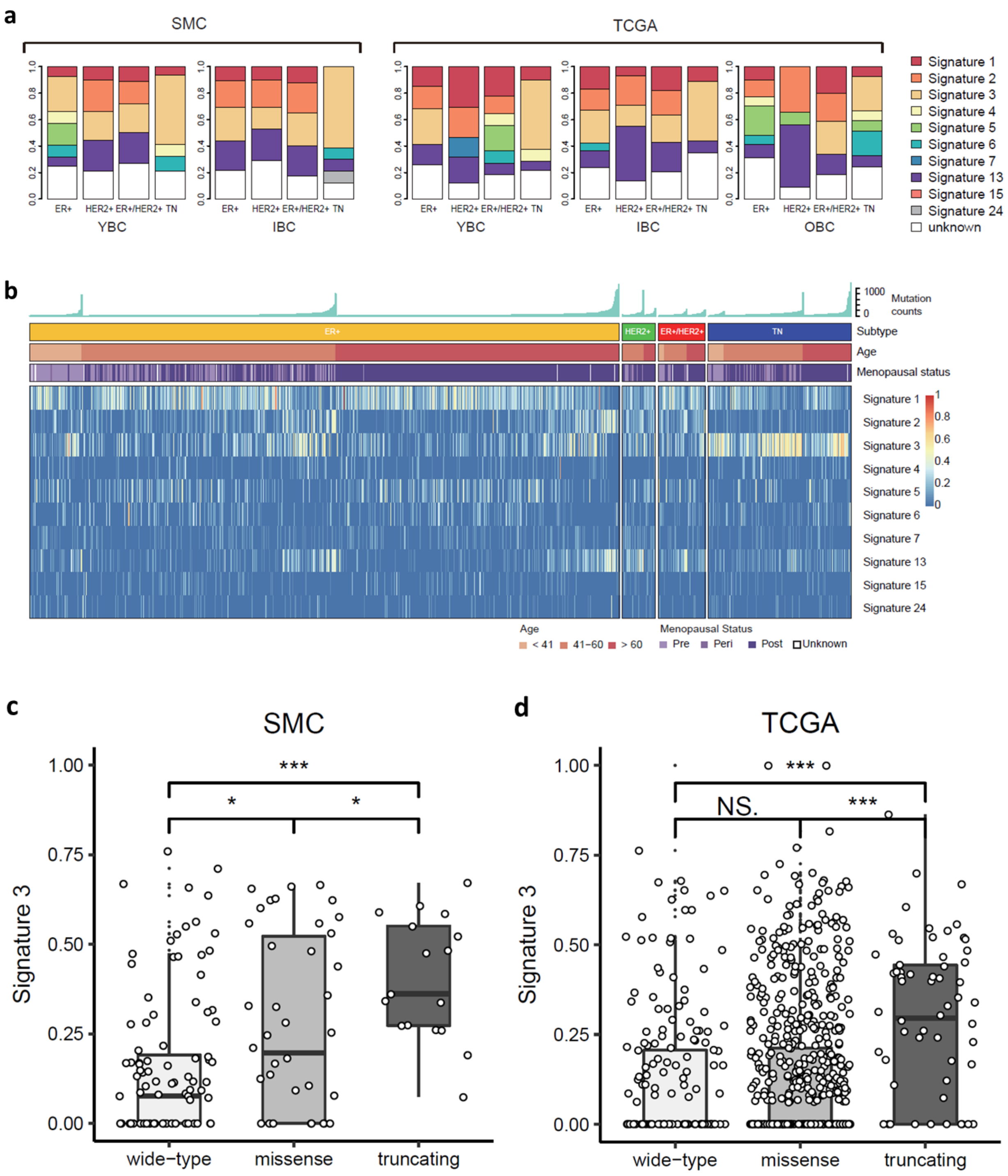

Supplementary Figure 5: Landscape of mutation signatures. (a) Mutation signature distribution across subtypes and age groups in SMC and TCGA. (b) Heatmap showing the scores of mutation signatures (rows) in TCGA samples (columns) grouped by molecular subtypes and age groups. Mutation count and menopausal status are also shown for individual samples. Pre: pre-menopausal. Peri: peri-menopausal. Post: post-menopausal. Boxplots comparing the distributions of signature 3 scores in SMC (c) and TCGA (d) samples grouped by BRCA1/BRCA2 germline mutation status - wild-type, mutated with ≥ 2 germline missense variants and mutated with protein-truncating variants. Significance levels from Student's t tests were indicated. The box is bounded by the first and third quartile with a horizontal line at the median and whiskers extend to the maximum and minimum value.

Supplementary Figure 6

a

Cohort	Sample Type	# Samples
SMC	Tumor	168
	Adjacent Normal	10
TCGA	Tumor	1,116
	Adjacent Normal	111
CCLE	Cell line	55
GTEX	Normal Tissue	218

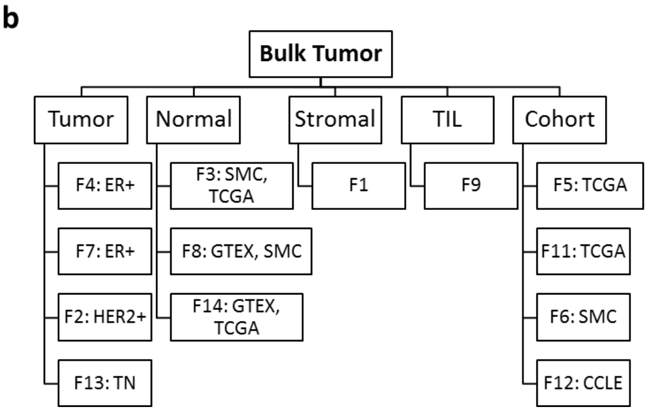

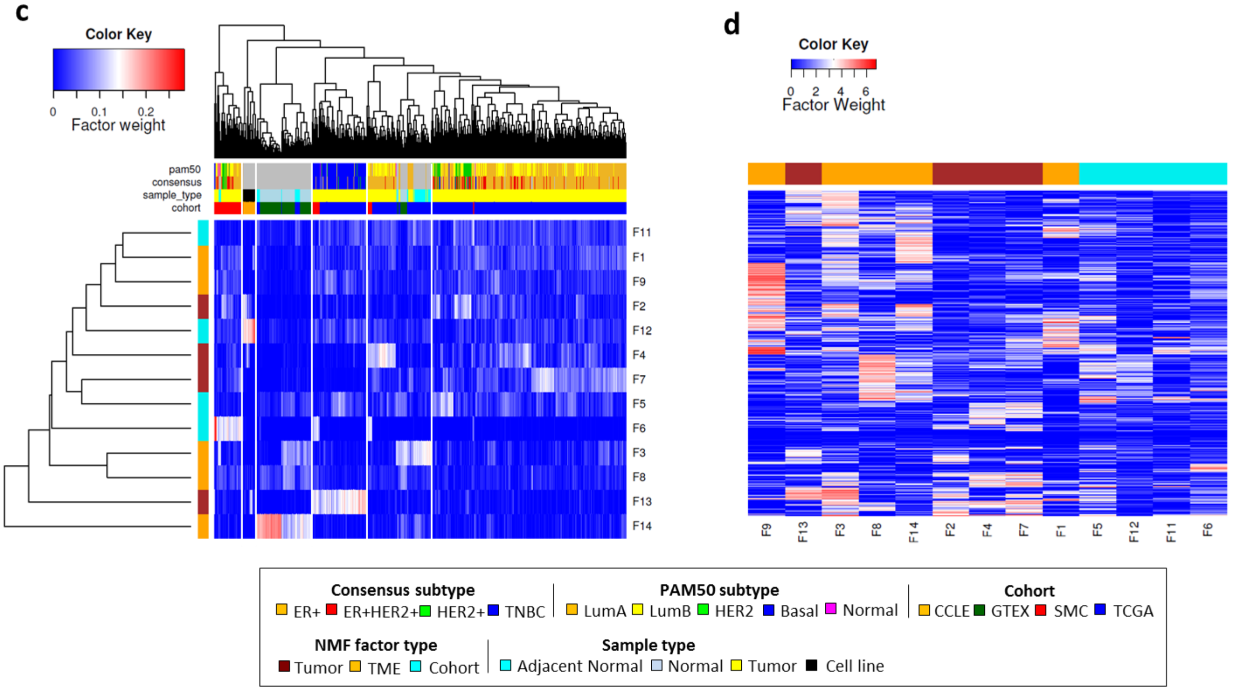

Supplementary Figure 6: Virtual tumor micro-dissection identified distinct tumor intrinsic and microenvironment factors. (a) Cohorts making up the expression compendium. (b) 13 NMF factors were attributed to 4 tissue compartments - tumor intrinsic, stroma, tumor infiltrating leukocyte (TIL) and normal tissue. For each factor, the highest scoring cohorts or subtypes are shown. Cohort: cohort specific factors. (c) Heatmap of sample weight matrix H with rows representing NMF factors and columns representing samples from the expression compendium. Column color labels represent molecular subtype classifications (Consensus and PAM50), sample type (Tumor, Adjacent normal, Healthy normal, cell line) and cohort (SMC, TCGA, CCLE and GTEx). (d) Heatmap of gene weight matrix W with rows representing factor genes and columns representing NMF factors. NMF factor type: tumor – tumor intrinsic, TME – tumor microenvironment, cohort – cohort specific.

Supplementary Figure 7

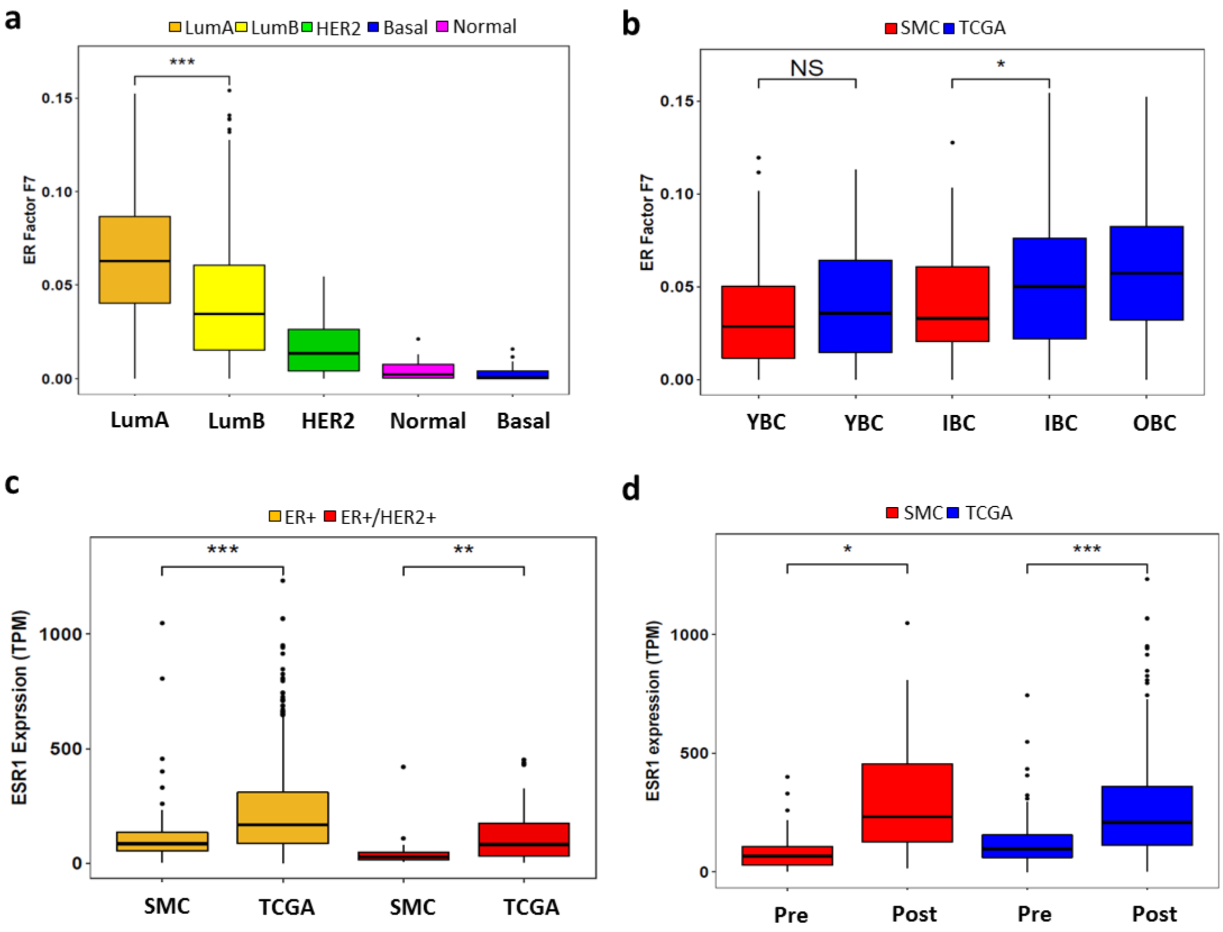

Supplementary Figure 7: Distribution patterns of ER associated factor and gene expression. Box plots showing the distributions of F7 factor weight across PAM50 subtypes (a) and age-based groups (b) in SMC and TCGA. Box plots showing the distribution of *ESR1* expression in TCGA vs. SMC across ER+ and ER+/HER2+ subtypes (c) and in pre-menopausal ER+ BCs compared to post-menopausal ER+ BCs in SMC and TCGA (d) *P*-value was determined by Student's t test: *** $p < 0.001$; ** $p < 0.01$; * $p < 0.05$; NS: $p \geq 0.05$. The box is bounded by the first and third quartile with a horizontal line at the median and whiskers extend to the maximum and minimum value.

Supplementary Figure 8

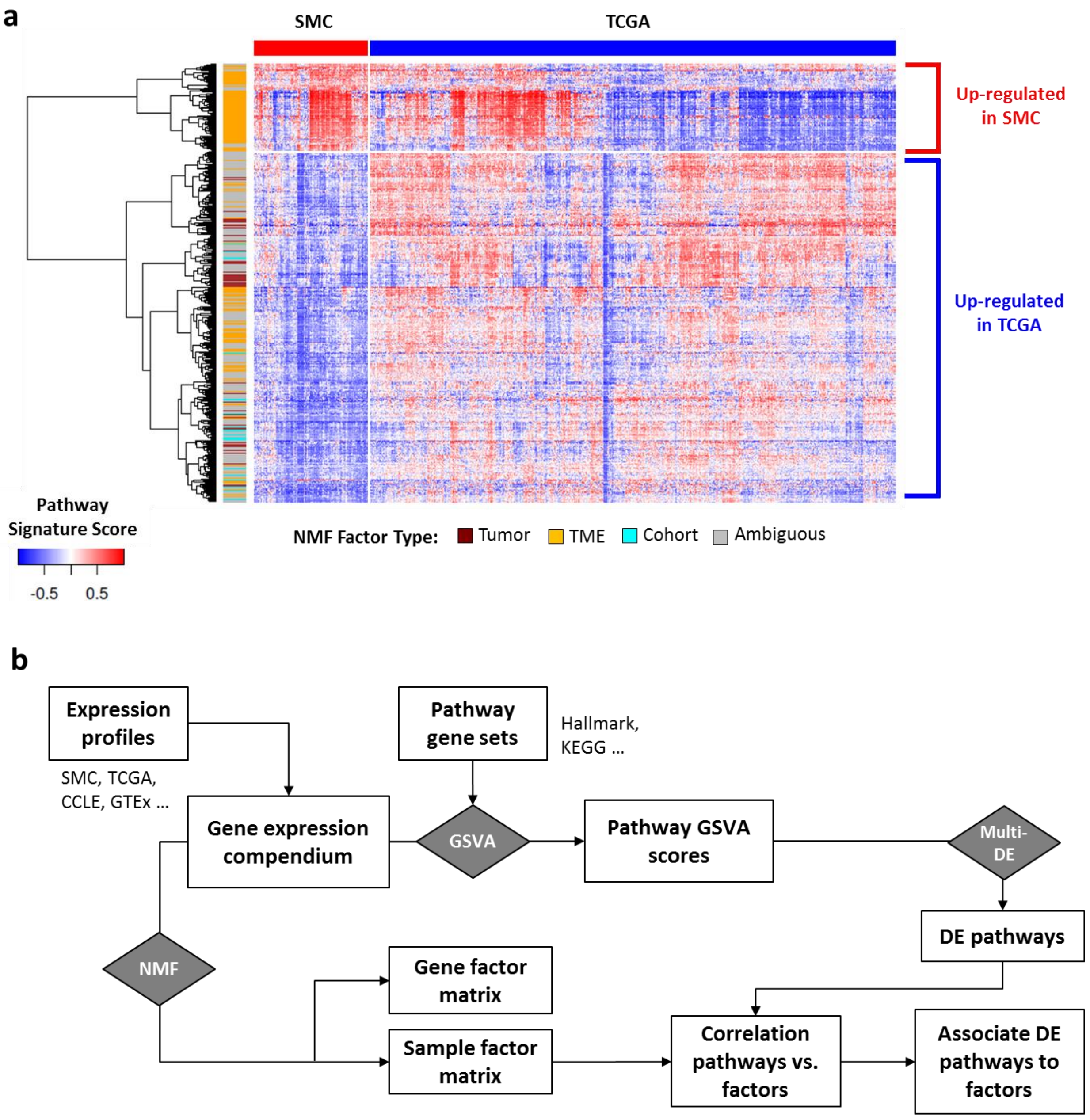

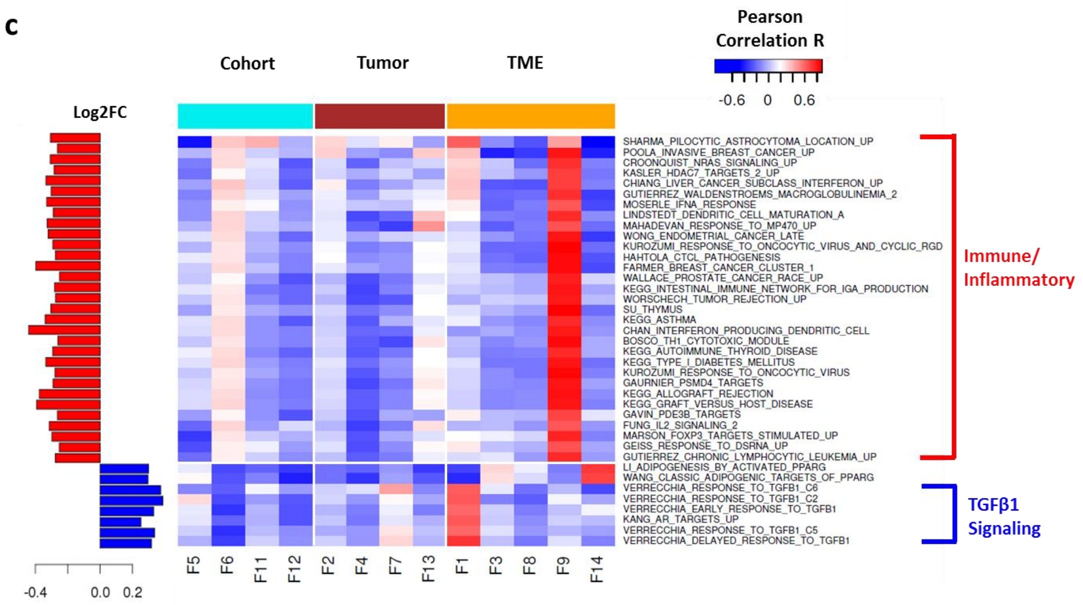

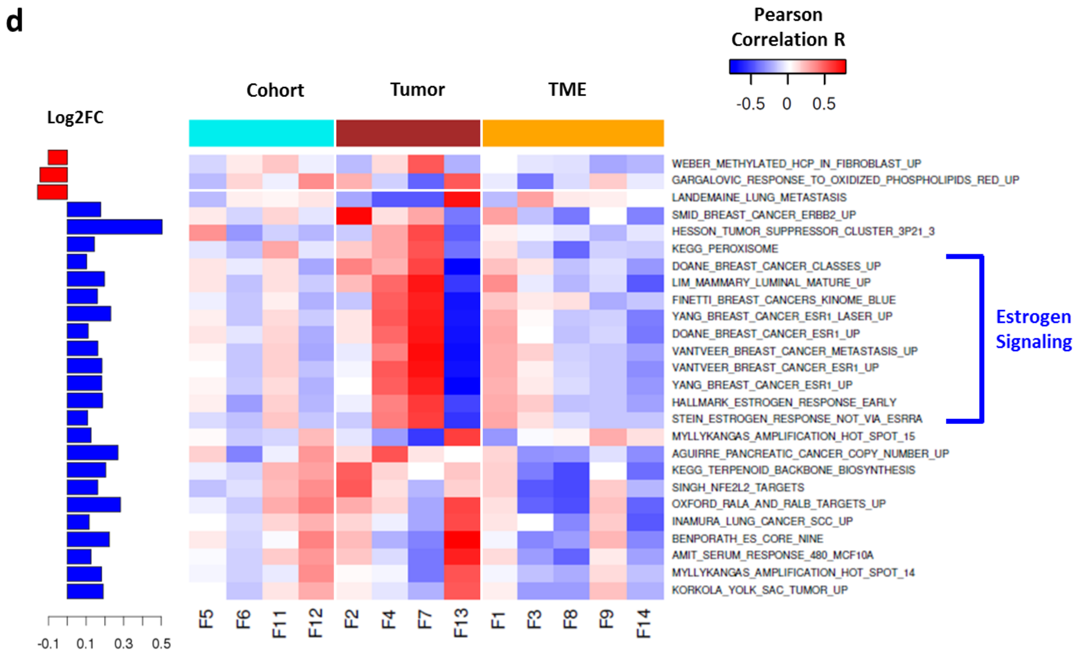

Supplementary Figure 8: Differential expression analyses of SMC pre-menopausal vs. TCGA post-menopausal tumors. (a) GSVA expression signature scores of DE pathways identified by comparing SMC pre-menopausal vs. TCGA post-menopausal tumors. Row color labels represent tissue compartmental origins associated with DE pathways. (b) Work flow to identify intratumoral compartmental origins for differentially expressed (DE) pathways through integrative analysis of pathway GSVA scores and NMF factor weight. Heatmap of correlation coefficients between DE pathways (rows) and NMF factors (columns) attributed to the TME (c) and tumor intrinsic (d) compartments. Bar charts to the left show the log2-fold-changes (log2FC) of DE pathways up-regulated (red) and down-regulated (blue) in SMC vs. TCGA. The following criteria were used to select gene sets as shown from the Hallmark, KEGG and C2 – chemical and genetic perturbations collections: Log2FC > 0.2; maximum r > 0.6 among TME factors (c) or maximum r > 0.5 among tumor factors (d).

Supplementary Figure 9

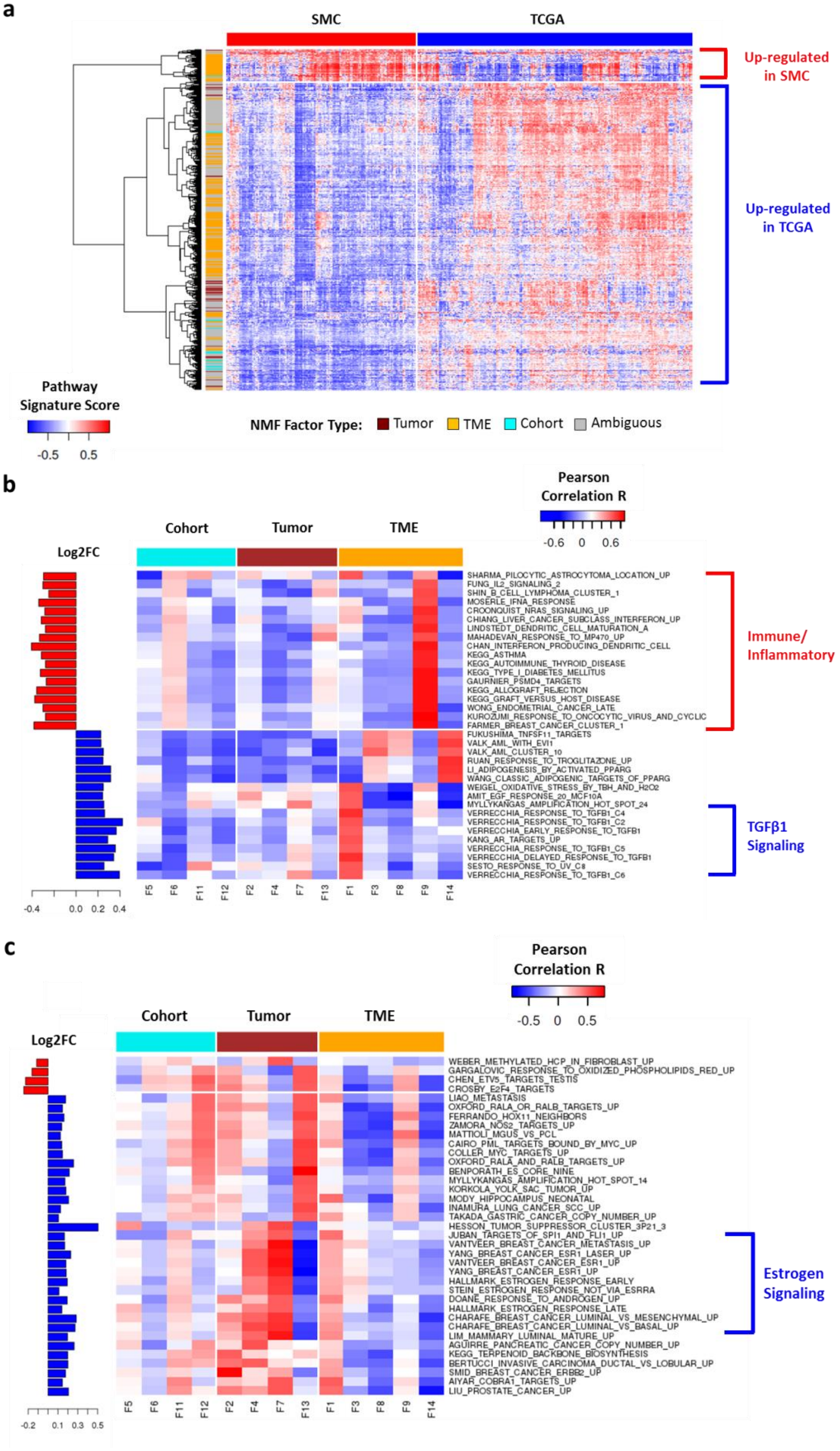

Supplementary Figure 9: Differential expression analyses of SMC pre-menopausal vs. TCGA pre-menopausal tumors. (a) GSVA expression signature scores of DE pathways identified by comparing SMC pre-menopausal vs. TCGA pre-menopausal tumors. Heatmap of correlation coefficients between DE pathways (rows) and NMF factors (columns) attributed to the TME (b) and tumor intrinsic (c) compartments. Bar charts to the left show the log2-fold-changes (log2FC) of DE pathways up-regulated (red) and down-regulated (blue) in SMC vs. TCGA.
