# Supplementary material for: Multi-omics profiling of younger Asian breast cancers reveals distinctive molecular signatures
Source: Nat Commun. 2018 Apr 30;9:1725. doi: 10.1038/s41467-018-04129-4 (PMC5928087; doi:10.1038/s41467-018-04129-4)
Supplement: Supplementary file 3 — Description of Additional Supplementary Files [file 41467_2018_4129_MOESM3_ESM.pdf]

Supplementary Data Legends

Supplementary Data 1: SMC and TCGA sample annotation.

Supplementary Data 2: Clinical data summary of SMC and TCGA.

Supplementary Data 3: Molecular subtype distribution in SMC and TCGA. This table contains proportions of molecular subtypes (Consensus, IHC and PAM50) in different age groups and cohorts. Also included are statistical significances of differential distribution between different sample groups.

Supplementary Data 4: *BRCA1/BRCA2* germline pathogenic mutations.

Supplementary Data 5: Mutation prevalence of cancer driver genes in SMC and TCGA. This table contains mutation frequencies (% samples) in different cohorts and age groups for significantly mutated genes. Also included are statistical significances of differential distribution and frequencies of germline pathogenic mutations for *BRCA1* and *BRCA2*.

Supplementary Data 6: Somatic mutation predictions in SMC. Detailed annotations for 6,885 somatic protein-altering mutations.

Supplementary Data 7: Significantly mutated genes. Significantly mutated genes identified by MutSigCV based on combined mutations from SMC and TCGA ("Combined"), SMC mutations alone ("SMC") and TCGA mutations alone ("TCGA"). n_nonsilent: number of protein-altering mutations. n_silent: number of silent mutations.

Supplementary Data 8: Somatic alteration prevalence of cancer driver genes in SMC and TCGA. This table contains the prevalence (% samples) of somatic alterations, including protein-altering substitutions, insertions, deletions, copy number amplifications and deletions, for frequently altered genes in different age groups and cohorts. Amplification is defined as absolute copy number ≥ 6 and deletion as copy number ≤ 1. Also included are statistical significances of different group comparisons. Both direct comparisons with Fisher's exact test and comparisons adjusting for tumor stage with logistic regression were performed. *P*-value was calculated using the Fisher's exact test and FDR corrected using the Benjamini-Hochberg method.

Supplementary Data 9: Somatic alteration prevalence of oncogenic pathways. This table contains alteration prevalence of five BC related oncogenic pathways in different age groups and cohorts. A pathway was altered in a sample if one or more genes in that pathway harbor alteration in the sample. Pairwise comparison of alteration prevalence was performed between different groups using the Fisher's exact test.

Supplementary Data 10: Somatic mutation signatures. (a) Mutation signature distribution across subtypes and cohorts. (b) Mutation signature correlation with mutation burden, number of protein-altering somatic mutations in each sample.

Supplementary Data 11: Pathway enrichment of NMF factors. Statistical significance for pairwise associations between DE pathways and NMF factors.

Supplementary Data 12: Differentially expressed genes and pathways. (a) Differentially expressed genes (a) and pathways (b) identified from two comparisons - SMC pre-menopausal vs. TCGA post-menopausal and SMC pre-menopausal vs. TCGA pre-menopausal.

Supplementary Data 13: Multivariate analyses of distinctive molecular features. Multivariate analysis results for distinctive molecular features and key clinicopathologic factors using samples from all subtypes and only TNBC.
